# MWCNTs-Reinforced Epoxidized Linseed Oil Plasticized Polylactic Acid Nanocomposite and Its Electroactive Shape Memory Behaviour

**DOI:** 10.3390/ijms151119924

**Published:** 2014-10-31

**Authors:** Javed Alam, Manawwer Alam, Mohan Raja, Zainularifeen Abduljaleel, Lawrence Arockiasamy Dass

**Affiliations:** 1 King Abdullah Institute for Nanotechnology, King Saud University, P.O. Box 2455, Riyadh 11451, Saudi Arabia; E-Mail: ldass@ksu.edu.sa; 2 Research Center, College of Science, King Saud University, P.O. Box 2454, Riyadh 11451, Saudi Arabia; E-Mail: malamiitd@gmail.com; 3 Amity Institute of Nanotechnology, Amity University, Sector 125, Noida 201303, U.P., India; E-Mail: mohanraja27@gmail.com; 4 Department of Medical Genetics, Faculty of Medicine, Umm Al Qura University, P.O. Box 715, Makkah Al Mukarramah 21421, Saudi Arabia; E-Mail: zainulbio@gmail.com

**Keywords:** shape memory effect, epoxidized linseed oil, hot press moulding, shape recovery, actuators

## Abstract

A novel electroactive shape memory polymer nanocomposite of epoxidized linseed oil plasticized polylactic acid and multi-walled carbon nanotubes (MWCNTs) was prepared by a combination of solution blending, solvent cast technique, and hydraulic hot press moulding. In this study, polylactic acid (PLA) was first plasticized by epoxidized linseed oil (ELO) in order to overcome the major limitations of PLA, such as high brittleness, low toughness, and low tensile elongation. Then, MWCNTs were incorporated into the ELO plasticized PLA matrix at three different loadings (2, 3 and 5 wt. %), with the aim of making the resulting nanocomposites electrically conductive. The addition of ELO decreased glass transition temperature, and increased the elongation and thermal degradability of PLA, as shown in the results of differential scanning calorimetry (DSC), tensile test, and thermo gravimetric analysis (TGA). Scanning electron microscopy (SEM) and atomic force microscopy (AFM) were used to observe surface morphology, topography, and the dispersion of MWCNTs in the nanocomposite. Finally, the electroactive-shape memory effect (electroactive-SME) in the resulting nanocomposite was investigated by a fold-deploy “U”-shape bending test. As per the results, the addition of both ELO and MWCNTs to PLA matrix seemed to enhance its overall properties with a great deal of potential in improved shape memory. The 3 wt. % MWCNTs-reinforced nanocomposite system, which showed 95% shape recovery within 45 s at 40 DC voltage, is expected to be used as a preferential polymeric nanocomposite material in various actuators, sensors and deployable devices.

## 1. Introduction

Polylactic acid (PLA) has been extensively studied to develop actuators for potential medical and industrial applications, due to its biodegradability, biocompatibility, renewability, and relatively low cost [1,2,3,4,5]. PLA is of special research interest in the field of actuator advances as it exhibits attractive thermo-mechanical properties and low glass transition temperature closely matched to body temperature [6,7,8,9]. On the other hand, it also suffers from an inherent problem of brittleness, which restricts its applications; particularly where a high percentage of elongation is required. In order to reduce its brittleness, many approaches, such as grafting, copolymerization, addition of plasticizers, polymers blending, and incorporation of nanofillers, have been employed in the literature [10,11,12,13,14,15]. Among them, a large number of investigations have been reported on blending PLA with various low molecular weight polymers, such as polyethylene oxide, polyethylene glycol, poly ε-caprolactone, polypropylene glycol, citrate esters, epoxidized vegetable oils, *etc.* [16,17,18,19,20,21]. However, the relatively high cost of the above-mentioned polymers has provided motivation for the use of low cost polymeric materials, such as epoxidized vegetable oils (EVOs). This substitution presents an interesting alternative, because not only are EVOs lower cost, but they offer other advantages, such as being a natural product, harmless, biodegradable, *etc.* Surprisingly, the presence of EVOs has seen distinct improvement in mechanical and thermal properties in their PLA blends [22,23,24,25]. Among the EVOs studied in the literature, epoxidized soybean oil (ESO)—a representative of EVOs—has been extensively reported as a plasticizer for PLA modifications [26,27,28,29]. Although demonstrating ESO plasticizing merits, ESO also showed several disadvantages, such as its leaching and migration from PLA end-products as a function of time. In general, a simple and common way to decrease the migration of plasticizers is to increase their molecular weight in such a way that retains their miscibility with the polymer matrix. Epoxidized linseed oil (ELO), as an attractive alternative to ESO, shows a high compatibility with PLA matrix, due to the availability of more epoxide groups. As we know, this epoxidation increases the polarity and the stability of vegetable oils by improving their compatibility with polymers, and provides a more energetic interaction to the matrix. Surprisingly, similar to ESO, ELO holds many outstanding properties, like good lubricity, low volatility, low odour, and good solvency for fluid additives. Several studies have been conducted on using ELO as a plasticizer and stabilizer in polymers; particularly for polyvinyl chloride (PVC), resulting in mechanical and thermal properties improvement in its PVC blend [30,31,32,33]. To the best of our knowledge, ELO has not yet been studied in modifying the properties of PLA and used as shape memory biomaterials to develop actuators.

In this present study, PLA was mixed with ELO to improve its flexibility. Then, multi-walled carbon nanotubes (MWCNTs) were incorporated into the ELO plasticized PLA matrix (the optimized PLA:ELO ratio used was 90:10) at three different loadings (2, 3 and 5 wt. %), with the aim of making plasticized PLA electrically conductive and suitable for electroactive-shape memory effect (electroactive-SME). The use of electricity, instead of other stimuli—such as temperature, chemicals, light, mechanical force, and magnetic field to activate the shape memory effect—was an interesting activation alternative towards a more homogeneous and effective heating in shape memory materials [34,35,36,37]. For this, the polymers are usually mixed with conducting fillers, such as carbon nanotubes (CNTs), short carbon fibers, carbon black, metallic Ni powder, conducting polymers, and so on [38,39,40,41,42,43]. Among these, CNTs have attracted a great deal of recent interest as conductive nanofillers to develop electrically conductive polymer nanocomposites. Hence, we prepared the electroactive shape memory polymer nanocomposite of ELO plasticized PLA and MWCNTs. Shape memory behaviour was then investigated using a fold-deploy “U”-shape bending test, using a DC power supply connected to an amplifier supplying a high DC voltage. The obvious changes in mechanical, thermal, electrical, and morphological properties of the resulting nanocomposite with the addition of both ELO and MWCNTs were also studied by typical analytical techniques, such as tensile testing, differential scanning calorimetry (DSC), thermo gravimetric analysis (TGA), scanning electron microscopy (SEM), and atomic force microscopy (AFM).

## 2. Results and Discussion

### 2.1. Morphology

**Figure 1:**
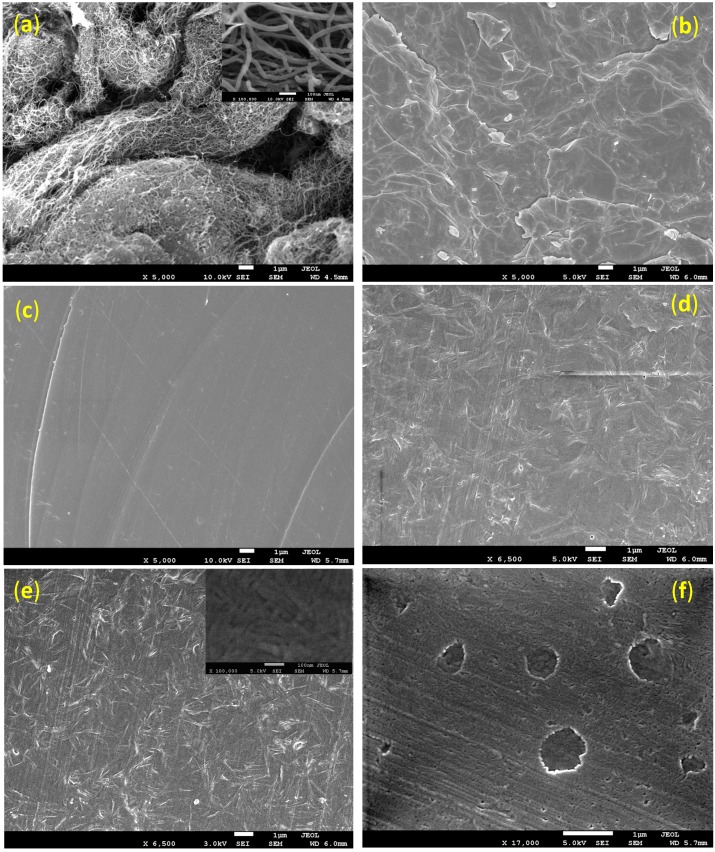
Scanning Electron Microscopy (SEM) images of (**a**) multi-walled carbon nanotubes (MWCNTs) as received; (**b**) neat polylactic acid (PLA); (**c**) epoxidized vegetable oils (ELO) plasticized PLA; (**d**–**f**) 2, 3 and 5 wt. % MWCNTs-reinforced PLA/ELO nanocomposites.

Figure 1 shows the surface morphology SEM micrographs of neat PLA, ELO plasticized PLA, and MWCNTs-reinforced PLA/ELO nanocomposites. As shown in Figure 1b, neat PLA presented an irregular and coarseness fracture surface, due to its brittle nature. However, as ELO was added into the PLA matrix, the morphology changed from brittle fracture surface to a smooth and uniform surface. Even though no aggregates and brittle cracks were observed, this morphology transformation evidences good interfacial adhesion or compatibility between PLA matrix and ELO. Furthermore, the incorporation of MWCNTs changed the morphology to be different from that of the neat PLA and plasticized PLA. As shown in Figure 1d–f, when the MWCNT’s content was 3 wt. %, the surface was continuous and dense. However, as the concentration of MWCNTs increased from 3–5 wt. %, interface defect like void formation, polymer bubbles, and a rigidified polymer layer around the MWCNTs were observed (see Figure 1f). These defects reflected the mechanical properties of the resultant nanocomposites (as can be seen in the tensile results). A continuous and dense surface formation, in the nanocomposite with a 3 wt. % loading of MWCNTs, could be attributed to uniformly dispersed MWCNTs; which provided a greater matrix/MWCNTs interfacial area and an enhanced matrix–MWCNTs interface contact. It is well-known that uniformly dispersed MWCNTs in a matrix serve as decent bridging elements, help to construct a surface with adequate features, and serve as barriers that avoid the growth of micro-cracks and void formation in the nanocomposites. AFM was used to better understand the surface and interface morphologies of the MWCNTs-reinforced nanocomposite samples as it is a promising technique for compositional and heterogeneity mapping in polymer nanocomposites.

**Figure 2:**
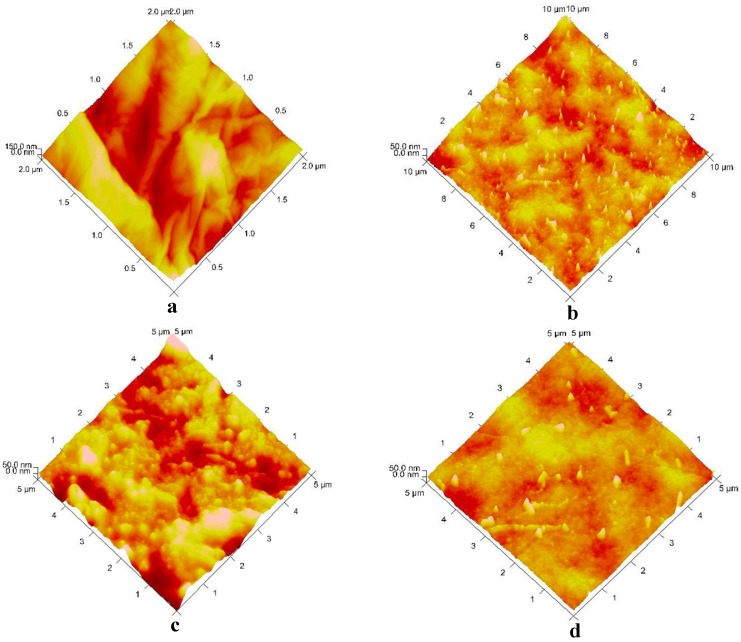
Atomic force microscopy (AFM) 3D images of (**a**) ELO plasticized PLA; (**b**–**d**) 2, 3 and 5 wt. % MWCNTs-reinforced PLA/ELO nanocomposites.

As shown in the AFM 3D images (Figure 2a–d), plasticized PLA exhibited a surface consisting of a ridge-and-valley structure. However, the addition of MWCNTs into the plasticized PLA matrix changed the surface topography from a ridge-and-valley structure to a regular with coarseness surface. Furthermore, as the MWCNT’s content increased, the surface was seen in a two-phase structure consisting of bright nodular domains and dark interstitial regions; thus indicating an inhomogeneous microstructure/heterogeneous structure of nanocomposites (see Figure 2d). Hence, the surface topographical study, done by AFM, shows that the bulk microstructure of MWCNTs-reinforced nanocomposite samples, with a high loading content of MWCNTs, was heterogeneous.

### 2.2. Thermal Properties

**Figure 3:**
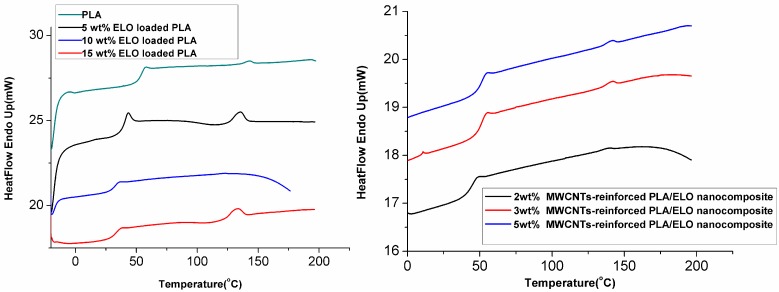
Differential Scanning Calorimetry (DSC) thermograms of ELO plasticized PLA, and MWCNTs-reinforced PLA/ELO nanocomposites.

Figure 3 shows the DSC thermograms of PLA, plasticized PLA with three weight ratios ELO (5, 10 and 15 wt. %), and MWCNTs-reinforced PLA/ELO nanocomposites. The thermal properties demonstrated that the addition of ELO resulted in a decrease in the Tg for plasticized PLA; even though the decrease in Tg was enhanced with a higher ELO content, due to the plasticizing effect. On the other hand, a slight increase in both Tg and Tm was observed when the MWCNTs were added to PLA. This was most likely due to the combined effect of increased interfacial adhesion between MWCNTs and the host matrix, and possible cross-linking between MWCNTs and the matrix, as both parameters are known to restrict the movement of chains of the host polymers.

Generally, the addition of plasticizers caused a decrease in the Tg of the host polymer; because plasticizers work by embedding themselves between the chains of the host polymer, spacing them apart (*i.e.*, increasing the “free volume”); and thus, making the polymers softer. Apart from this, the DSC curves exhibited one Tg value, indicating that ELO was compatible with the PLA matrix and could therefore be used as a potential plasticizer for PLA modification.

**Figure 4:**
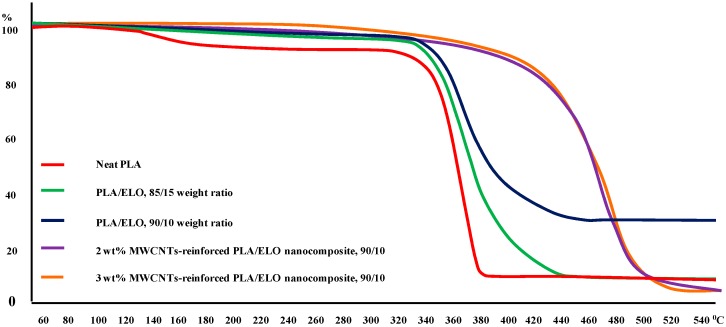
Thermo gravimetric analysis (TGA) curves for neat PLA, PLA/ELO (85:15), PLA/ELO (90:10), MWCNTs-reinforced PLA/ELO nanocomposites.

Figure 4 shows the TGA curves of PLA, plasticized PLA, and MWCNTs-reinforced PLA/ELO nanocomposites. Significant changes were observed in the characteristic thermal temperature’s onset temperature, which is the initial weight loss temperature, and the maximum degradation temperature, which is the highest thermal degradation rate temperature. As can be seen, the pure PLA exhibited an onset temperature of 321.9 °C and a degradation temperature of 380.7 °C, which increased to 362.3, and 435.7 °C, respectively; once ELO was incorporated into the PLA matrix. However, upon the addition of MWCNTs into the ELO plasticized PLA matrix, a remarkable change in the onset degradation temperature was observed. Plasticized PLA showed an onset temperature of 362.3 °C, which increased to 460.3 and 462.4 °C when 2 and 3 wt. % of MWCNTs, respectively, was added into the plasticized PLA.

### 2.3. Mechanical Properties

**Figure 5:**
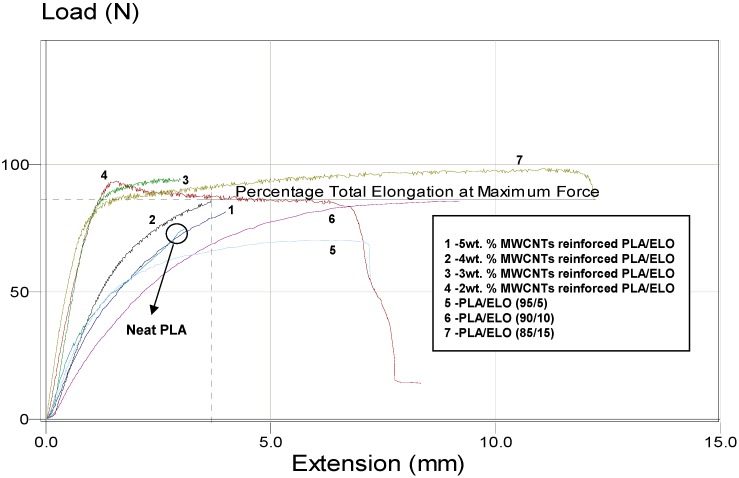
Load-Extension curves for Neat PLA, ELO plasticized PLA, and MWCNTs reinforced ELO plasticized PLA nanocomposites.

Figure 5 shows the load-extension curves of neat PLA, plasticized PLA with different weight ratios (5, 10 and 15 wt. %) of ELO, and MWCNTs-reinforced PLA/ELO nanocomposites obtained by tensile tests at room temperature. As the results show, neat PLA exhibited the characteristics of a glassy polymer with low tensile elongation. The glassy behaviour of PLA changed when ELO was added; even when the concentration of ELO was increased, the glassy behaviour (or the tensile elongation) of PLA linearly decreased; thus explaining the plasticizing effect of ELO. However, the addition of ELO caused a decrease in the load at break; likely due to decreased intermolecular forces between the polymer chains.

Generally, plasticizers are known to make a polymer matrix softer and more flexible by decreasing the intermolecular forces between the chains of the host polymer; thus, the polymer may elongate/stretch easier through applied loads. However, the tensile test results showed a drastic change in mechanical properties when the MWCNTs were incorporated into the plasticized PLA. As shown in the load-extension curves, the MWCNTs had an increased load at break; however, the incorporation of MWCNTs decreased the percentage of elongation in the resulting nanocomposites. As the results show, maximum extension and load at break increased to an optimum of 12.3 mm and 93 N, respectively, corresponding to the 3 wt. % of MWCNTs-reinforced nanocomposite system. This observed improvement in load at break could be accredited to the improved MWCNTs-matrix interaction, caused by homogeneously dispersed MWCNTs within the nanocomposite. However, as the MWCNT’s content increased, the mechanical properties diminished due to an increased agglomeration of MWCNTs in the nanocomposite as the agglomerated MWCNTs decreased the MWCNTs-matrix interactions and hindered load transfer across the MWCNTs-matrix interface. Thus, the resultant nanocomposites, at a higher amount of MWCNTs, exhibited poor mechanical properties. It is reasonable to conclude that the mechanical properties of MWCNTs-reinforced polymeric nanocomposite systems are mostly governed by the dispersion level of MWCNTs in the nanocomposite matrix.

### 2.4. Electroactive Shape Memory Effect

**Figure 6:**
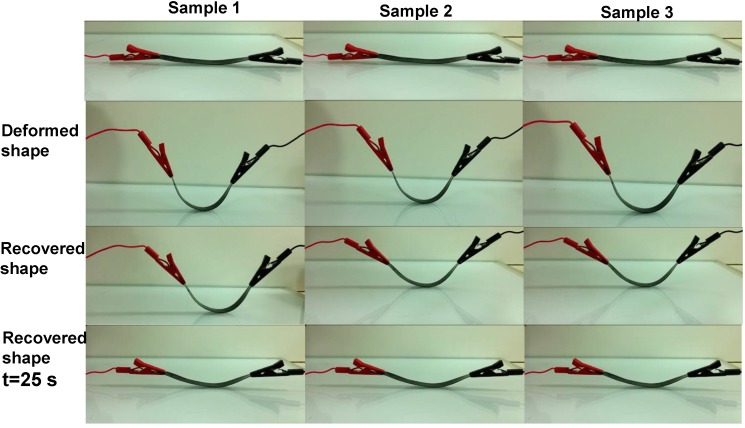
Shape recovery at DC voltage 40 V for the 2, 3 and 5 wt. % MWCNTs reinforced ELO plasticized PLA nanocomposites.

**Figure 7:**
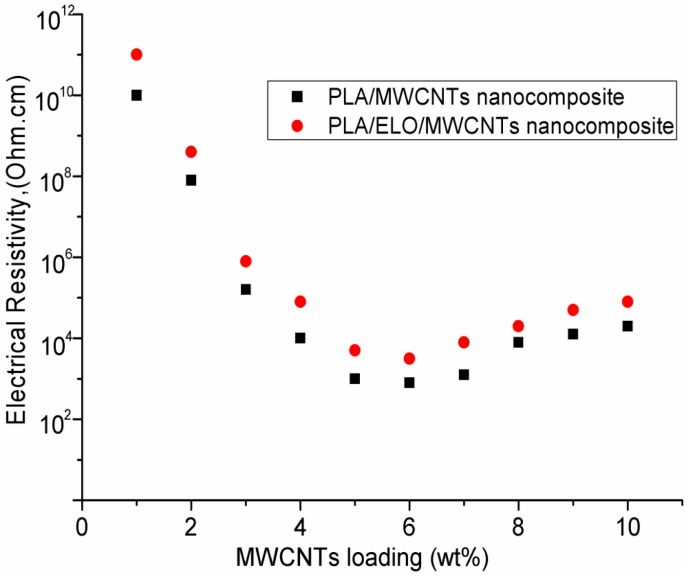
Electrical resistivity of MWCNTs reinforced PLA, and MWCNTs reinforced ELO plasticized PLA nanocomposites.

Figure 6 shows the electroactive-SME in MWCNTs-reinforced PLA/ELO nanocomposites as a function of MWCNT’s content. From the results, it was observed that the extent of electroactive-SME in the resulting nanocomposites was strongly affected by the added content of MWCNTs. As can be seen, the nanocomposite (made from 3 wt. % MWCNTs) showed a recovery of 95% within 45 s; whereas a similar recovery level took 85 s when MWCNTs concentration was increased from 3–5 wt. %. However, the nanocomposite with a MWCNTs loading below 3 wt. %, showed a slow recovery (75 s was required to reach a recovery of 95%), which indicates that an MWCNTs loading below 3 wt. % could not form enough continuous conductive networks throughout the nanocomposite; and thus, induced poor electrical conductivity properties in the nanocomposite that were responsible for a slow activation of shape recovery by applying voltage (see Figure 7). A slow recovery, upon a high loading of MWCNTs, can be explained by the increased formation of MWCNTs clusters in the nanocomposites; as well as the formation of non-continuous electrical conductive networks in the nanocomposite, caused by the agglomeration of MWCNTs that decreased the electrical conductivity properties (as shown in Figure 7). A slow shape recovery at high loading levels (more than 3 wt. %) of MWCNTs could be accredited to the increased stiffness of MWCNTs-reinforced PLA/ELO nanocomposites; due to the formation of more cross-linked structures in the nanocomposite, which are prone to have negative effects on shape recoverability (as shown in the tensile test results (Figure 5), load-elongation curve, nanocomposites with high loading of MWCNTs showed a higher stiffness than the nanocomposites with low loading levels of MWCNTs). We may conclude that the optimum electroactive-SME, in the 3 wt. % MWCNTs-reinforced nanocomposite system, is accredited to a better MWCNTs-matrix adhesion, caused by a uniform dispersion of the MWCNTs throughout the nanocomposite, which decreased the interfacial electrical resistance within the nanocomposite and led to a more efficient heat transfer throughout the nanocomposite.

The 3 wt. % MWCNTs-reinforced system, which showed a 97% recovery at a 40 DC voltage, is expected to be used as the preferred polymeric nanocomposite material in various actuators, sensors and deployable devices.

## 3. Experimental Section

### 3.1. Materials

Polylactic acid (PLA) commercial grade PLA-05, with 1.25 g/cm^3^ density (Grafen Chemical Industries Co. (Ankara, Turkey)), and multi-walled carbon nanotubes (Nanocyl^®^-7000 with average diameter 9.5 nm; length = 1.5 μm; purity > 99 wt. %) were purchased from Nanocyl Korea Ltd., Seoul, South Korea. Epoxidized linseed oil (Shankar Dyes and Chemicals, New Delhi, India, with oxirane oxygen 9.3 g/100 g; EEW g/equivalent 171; Viscosity 800 mPa·s at 25 °C; Density 1.03 g/mL), chloroform CHCl_3_ 30%, methanol 99.8%, acetone 99.9% (Alfa Aesar-Johnson Matthey Company, Hyderabad, India) were used as received.

### 3.2. MWCNTs-Reinforced Epoxidized Linseed Oil (ELO) Plasticized Polylactic Acid (PLA) Nanocomposite Preparation

Initially, ELO plasticized PLA-nanocomposite matrix was prepared by solution blending, and chloroform (CHCl_3_) was used as a solvent. During the blending, PLA (10 g) was placed into a 250 mL volume three-necked flask that was equipped with a mechanical stirrer, and 80 mL of chloroform was added and stirred at a slow speed (between 55–75 rpm) at 25 ± 1 °C. After PLA solubilisation, ELO of 5%, 10% and 15% of PLA weights were added and stirred for 3 h to form a PLA/ELO blend solution. Next, MWCNTs, mixed with CHCl_3_, were placed into an ultrasonic bath for 10 min to remove any aggregates and to ensure a better dispersion in the CHCl_3_. It was then incorporated into the as-prepared PLA/ELO blend solution and the mixtures were allowed to stir again at a temperature of 25 ± 1 °C. A uniform dispersion of MWCNTs was achieved by the repetition of ultrasonication and slow stirring. Finally, the freshly prepared MWCNTs-reinforced ELO plasticized PLA nanocomposite solution was poured into a glass substrate frame, having a boundary wall of 5 mm. On evaporation of the CHCl_3_, a flexible, non-fractured sheet was formed, which was kept in a vacuum oven for 1 h at 35 °C. This permitted the CHCl_3_ within the samples to completely evaporate. However, the thickness of the prepared nanocomposite film was not uniform. To make it uniform, the prepared sheet was again processed in a hydraulic hot-press, by keeping the prepared sheet between aluminium foils with an applied load of 2 kN at 65 °C for 10 min. They were then cooled to room temperature to form sheets of a uniform thickness (0.45 ± 0.01 mm).

### 3.3. Characterizations

#### 3.3.1. Scanning Electron Microscopy (SEM)

SEM (JEOL, Akishima-Shi, Japan, was used to study the morphology of the samples by image analysis of the surface texture. For the SEM study, clean, dry and dust-free samples were mounted on sample holders (called specimen stubs) using double-sided conductive adhesive tape. They were then placed in a vacuum chamber for sputtering, in order to make the samples electrically conductive. An acceleration voltage of 5 kV was used to study the surface morphology of the samples.

#### 3.3.2. Atomic Force Microscopy (AFM)

AFM provides three-dimensional (3D) images useful for understanding the surface structure of a sample. AFM (Veeco MultiMode SPM, New York, NY, USA) with a Nanoscope V controller, was used to measure the surface topography of the samples. 3D images were taken in the non-contact AFM imaging mode under ambient conditions at a scan frequency of 0.999 Hz. For the AFM study, a square-shaped sample (3 mm × 3 mm) was cut and glued onto a metal substrate. The surfaces that we examined were at scan sizes of 10 μm × 10 μm, 5 μm × 5 μm and 2 μm × 2 μm.

#### 3.3.3. Tensile Test

The mechanical properties of the samples were measured by tensile test. All tests were conducted at 25 ± 1 °C on Lloyd-LR5KPlus (Lloyd Materials Testing, West Sussex, UK) with a load cell capacity of 5 kN. Five replicates of each sample were employed for tensile testing. For the test, a dumbbell-shaped specimen (thickness of 0.5 mm, width of 12.7 mm, base length 127 mm and a gauge length of 25 mm) was prepared. A dumbbell-shape was used because it can be gripped within the jaws of the testing machine. The test was performed at a crosshead speed of 10 mm/min. Tensile data was analysed using NEXYGENPlus software.

#### 3.3.4. Thermal Analysis

The thermal properties of the samples were studied using differential scanning calorimetric (DSC, 6000 PerkinElmer, Santa Clara, CA, USA) and thermo gravimetric analysis (TGA, Mettler Toledo, Greifensee, Switzerland). The thermal events that we studied involved glass transition temperature (Tg) and melting temperature (Tm), and were analysed using PYRIS Manager Software. For the DSC analysis, samples were heated, from 20 to 180 °C at a heating rate of 40 °C/min, to remove any thermal history on the material. The samples were then cooled to −20 °C at a rate of 40 °C/min. A second heating took place at a heating rate of 10 °C/min from −20–200 °C using nitrogen gas with a flow rate of 5 mL/min. The thermal stability of the samples was analysed using Mettler Toledo; model TGA 2050. Samples (10 mg) for the TGA study were placed inside aluminium pans and heated under flowing nitrogen (10 mL/min) ranging from 25–550 °C at 10 °C/min, in order to obtain the corresponding thermal decomposition profiles.

#### 3.3.5. Electroactive-Shape Memory Experiment

Electroactive-SME, which is the ability of a material to be severely deformed into a temporary shape and then returned to its original shape, simply by Joule heating induced by passing an electric current through samples, was performed using a fold-deploy “U”-shape bending test. For this, a sample (sized 9 mm × 50 mm × 0.45 mm) was deformed into a “U” shaped structure by keeping it in hot water for 10 min at 85 °C, then immediately placed into cold water for 1 min, to fix it into the intended shape. Next, the sample was taken out of the cold water and the deforming stress was released. No free recovery was observed at ambient temperature. The electroactive-SME in the samples was analysed. For this, one edge of the deformed sample was wrapped with an electrically conductive carbon tab and connected to the probes of an amplifier supplying a DC voltage; Keithley 2400, Ohio, OH, USA. All measurements were made at room temperature. Electroactive-SME at a DC voltage of 40 V was studied as a function of time, and shape recovery was captured using a digital camera. The percentage of shape recovery was calculated using the following Equation:

*R* (%) = (θi − θr)/θi × 100 (1)

where, *R* is the % shape recovery, and θi and θr are the deformation angles before and after shape recovery, respectively.

## 4. Conclusions

A novel electroactive shape memory polymer nanocomposite, made of ELO plasticized PLA, and three different weight percentages (2, 3 and 5 wt. %) of MWCNTs, were prepared using a combination of solution blending, solvent cast technique, and hydraulic hot press moulding. As the results show, the extent of electroactive-SME in the resulting nanocomposites was strongly affected by the added content of MWCNTs. A nanocomposite, made using a 3 wt. % MWCNTs loading, showed a shape recovery of 95% within 45 s. Meanwhile, a similar recovery level took 85 s when the MWCNT’s concentration was increased from 3–5 wt. %. However, a nanocomposite made using a MWCNTs loading below 3 wt. % showed a slow recovery of 75 s to reach a similar recovery level. Tensile, DSC, and TGA results revealed that the incorporation of ELO improved the mechanical and thermal properties of PLA. Furthermore, the Tg of PLA decreased, almost linearly, with an increase in the ELO content, indicating that ELO significantly plasticized the PLA.
